# Current Concepts in Pediatric Robotic Assisted Pyeloplasty

**DOI:** 10.3389/fped.2019.00004

**Published:** 2019-01-24

**Authors:** Ramphis A. Morales-López, Marcos Pérez-Marchán, Marcos Pérez Brayfield

**Affiliations:** ^1^Division of Urology, Department of Surgery, University of Puerto Rico School of Medicine, San Juan, PR, United States; ^2^HIMA-San Pablo Group, Caguas, PR, United States

**Keywords:** pediatric urology, robotics, pyeloplasty, UPJ obstruction, children

## Abstract

Robotic surgery in pediatric urology has been gaining popularity since its introduction almost two decades ago. Robotic assisted pyeloplasty is the most common robotic procedure performed in pediatric urology. Advances in robotic technology, instrumentation, patient care and surgical expertise have allowed the correction of ureteropelvic junction (UPJ) obstruction in most patients using this minimally invasive technique. The excellent experience with robotic assisted pyeloplasty has challenged other approaches as a new standard for the treatment of UPJ obstruction. In this review, we will describe the technique as it relates to the different robotic platforms, review the surgical experience and compare its results to other surgical approaches. Also, we will discuss patient and parent satisfaction, cost and financial considerations, along with evaluating the future of robotic surgery in the treatment of UPJ obstruction.

## Introduction

Despite its recent beginnings, robotic assisted surgery has been progressing in the treatment of many conditions in pediatric urology. Since the introduction of laparoscopic pyeloplasty in 1993 in adults and 2 years later in the pediatric population, minimally invasive laparoscopic approach for the treatment of ureteropelvic junction obstruction (UPJO) became an evident viable option. In 1994, the first robotic system used in the urological practice known as AESOP was introduced. Later, the evolution of these devices would bring the Zeus system and finally the Da Vinci system while continuously increasing their precision and effectivity (1). This new surgical approach was embraced by doctors throughout the US and promoted a statistical increase in use throughout the country (2).

When compared to classical laparoscopic surgery, robotic assistance offers several benefits. Tremor cancellation, three-dimensional vision and 7° of freedom allow the surgeon to optimally perform in confined working spaces such as those found during pediatric surgery while executing precise and delicate movements with ease (3). In 2002, the first pediatric robotic procedure performed was the robotic laparoscopic pyeloplasty (4). The high incidence of UPJO combined with the surgeons' previous experience with the laparoscopic approach naturally made it a pioneer procedure for robotics in pediatric urology.

Until now, the gold standard method for treating UPJO is open dismembered pyeloplasty with a success rate between 90 and 100%. Laparoscopic pyeloplasty had gain popularity but it struggled to be adopted by many pediatric urologists because of its technical difficulty and tedious learning curve. However, robotic assisted laparoscopic pyeloplasty (RALP) had all the advantages of the laparoscopic approach with an ease of use and a much shorter learning curve. This allows some surgeons to transition from open pyeloplasty to a minimally invasive robotic approach without any previous laparoscopic experience. RALP has been the most commonly reported robotic procedure in children to date (5). In this review, we will describe the technique as it relates to the different robotic platforms, review the surgical experience over the last 5 years and compare its results to other surgical approaches. Also, we will discuss patient and parent satisfaction, cost and financial considerations, along with evaluating the future of robotic surgery in the treatment of UPJ obstruction.

## Background

UPJO is a common cause of pediatric hydronephrosis occurring in 1 per 1,000–2,000 newborns (6). Widespread use of antenatal ultrasonography (US) and the increase availability of postnatal imaging have resulted in earlier and more frequent diagnosis of hydronephrosis. UPJO is found more commonly in boys than in girls with up to 67% of cases involving the left kidney, and up to 10% seen bilaterally (7). Renal dysplasia, multicystic dysplastic kidney, duplicated renal collecting system where the lower pole UPJ is usually the obstructed segment; horseshoe kidney; and ectopic kidney have been found in association with UPJO. The etiology can be described as lesions that involve the UPJ intrinsically, lesions that are extrinsic or a combination of both.

The initial postnatal evaluation is performed with a renal/bladder US in order to determine the presence of pelvocalyceal dilation with or without renal cortical thinning. The most widely used grading systems of the severity of hydronephrosis on US are the Society of Fetal Urology (SFU) system and the Anterior/Posterior (AP) diameter of the renal pelvis. In 2014, a multidisciplinary consensus group developed the urinary tract dilation (UTD) classification system pertinent to antenatal and postnatal evaluation. The new classification incorporated the following six US parameters: AP renal pelvis diameter (APD), calyceal dilation, renal parenchymal thickness, renal parenchymal appearance, bladder abnormalities, and ureteral abnormalities (8).

Diuretic renography is the most widely used non-invasive technique to determine the severity and functional significance of UPJ obstruction (9). Technetium-99 m mercaptoacetyltriglycerine (^99m^Tc-MAG3) is the ideal tracer for the pediatric population. One of the most useful measurements in diuretic renography is the estimate of differential renal function. This is considered significant when it is <40%. This percentage usually is well-correlated with the half-life (T1/2) washout curve.

Other than US and MAG3 renal scan there are other imaging and diagnostic tests also less commonly utilized for diagnosis of UPJO. Magnetic resonance imaging has been used by some center as their study of choice to evaluate UPJO (10). Developments in magnetic resonance imaging (MRI) technology have made it possible to image kidneys while assessing anatomy, renal transit times as well as intracellular metabolic parameters independent of blood flow and tubular function.

Indications for surgical interventions are ipsilateral UPJO with <40% of differential renal function (DRF) on diuretic renography, bilateral severe UPJO with renal parenchymal atrophy, obstructive pattern on diuretic renography with abdominal mass, urosepsis, or cyclic flank pain with or without vomiting and recurrent UTI under antibiotic prophylaxis.

### Robotic Assisted Laparoscopic Pyeloplasty (RALP)

RALP is now a well-established method of correcting UPJO (5). It has the advantage of being able to help overcome the difficulties encountered with laparoscopic dissecting and suturing. The basic principle is similar to that of laparoscopic pyeloplasty but facilitated by 3-D imaging and the help of an articulated instrument. The operative technique has evolved to the point where RALP can be successfully performed in most pediatric patients. Some limitations could be encountered in infants <5 mo. or patients with a small abdominal cavity due to the limited available working space.

### Positioning

The procedure starts with a crucial element for success: proper positioning of the patient. The patient is positioned at the edge of the table with the arms resting on a folded arm board. This will allow the robot arms to use their full range without table interference. Our preferred patient position is the 45° or modified flank position. This position with the use of the table rotation allow access and trocar placement in a near supine position avoiding potential complications. Also, this position allows for the intraabdominal contents to move away from the surgical site. We use two large jelly rolls to support the patient's back with the upper arm placed across the body in a praying position. The lower leg is bended at a 90° angle and the upper leg is straight. It is of outmost importance that the patient is well-padded specially between the legs, arms, and face. The patient is secured to the table with wide tape across the arms and shoulders, chest, hip, knee, and ankles. Care is taken not to place tape directly on patient's skin. With the help of the anesthesiologist, proper positioning, and adequate access to the patient's airway and IV lines are confirmed before the start of the procedure.

### Initial Access, Insufflation, and Trocar Placement

**Figure 1:**
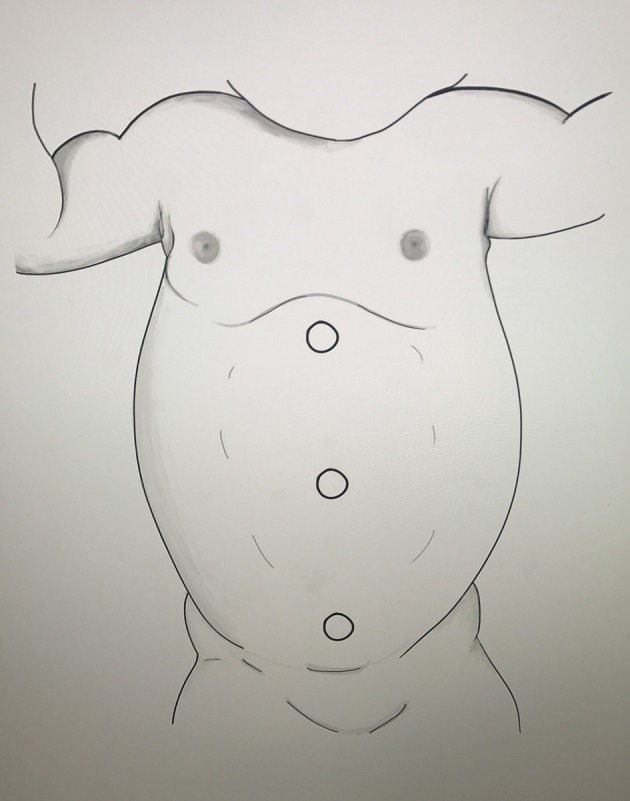
DaVinci Xi port placement for RALP (Drawing by Carla Fernandez).

With the table tilted away from the surgeon' side, the access is performed on a nearly flat patient. Our preferred approach is percutaneous using the Veress needle. Others have preferred the open Hassan technique for smaller children (11). After CO_2_ insufflation to 8–10 mmHg, a 5 mm optical trocar is placed under direct vision in the infraumbilical position allowing easy and safe access. The port position for robotic assisted laparoscopic pyeloplasty will be in straight line for most patients using the DaVinci Xi system (Figure 1). The 8 mm robotic trocars are placed under direct vision with the last trocar replacing the infraumbilical trocar. With the DaVinci Xi, the trocar should have at least 3 cm of separation in order to avoid robotic arms collision. Other have described the best trocar position for the Si system which needs to be modified depending on the patient's size. Several options include straight line, triangulation, and HIDES (12). The HIDES trocar positioning allows for better cosmetic results with an infraumbilical port and 2 additional lower abdominal ports. “Burping” of all ports will give additional intraabdominal space needed in order to successfully perform the procedure on smaller children.

### Docking With Si and Xi Systems

RALP requires the Xi robotic system to be docked at a 90° angle to the patient at the level of the infraumbilical port (camera site). We preferred to keep the robot at an established position and rotate the arms of the robot to adjust to the surgical site without moving the operating table. This allows the head of the patient to remain in the standard position and close to the anesthesiologists. Alternatively, the table could be rotated 180° to allow docking without rotation of the robotic arms. With the Si robotic system, the robot will need to be repositioned to come in a straight line with the camera site, trocar and the surgical site. As an example, the HIDES port placement the robot will dock from the inferolateral position (13).

### Surgical Technique and Stent

Cystoscopy with a retrograde pyelogram can be performed selectively at the start of the procedure. Indication for cystoscopy with retrograde pyelogram included complex anatomy (ectopic/horseshoe kidney) and need to clarify preoperative testing. Our preferred surgical technique for the corrections of UPJO is the dismembered pyeloplasty. The procedure follows the same surgical principles described in the laparoscopic pyeloplasty (14). Instruments used during the procedure include: 2 dissecting forceps, scissors, 2 needle drivers, and a suction device. The approach to the UPJ area can be transmesenteric for left sided UPJO or with medial mobilization of the colon for Right sided UPJO and with selected complicated left sided UPJO. Caution is needed during the initial dissection of the UPJ area to avoid injury to a lower pole crossing vessels. Tethered stitches using 3-0 prolene on a CT needle can be placed to the renal pelvis and proximal ureter to help with exposure and ease of the operation. We preferred to place a stent in all patients undergoing robotic assisted pyeloplasty. The stent can be placed in an antegrade or retrograde fashion depending on the surgeon's preference.

### Postop Care and Follow up

Indwelling urethral catheter is removed on POD # 1. Most patients are discharged within 24 h if they are able to void, tolerate diet and have adequate pain control. The ureteral stent is removed 6 weeks after the operation. Renal and bladder ultrasound is performed at 2 weeks., 3 mo., and 6 mo. post operatively. MAG-3 renal scan is reserved for symptomatic patients or significant residual hydronephrosis after 3 mo. follow up. Asymptomatic patients with residual hydronephrosis and good renal interval growth are followed with a renal and bladder ultrasound until resolution.

## Experience and Results

We retrospectively reviewed our experience with RALP using the DaVinci Xi robotic platform. We identified 41 patients with a mean age of 10.9 years (7 mo.−17 years). Ten patients were <1 year of age. Left RALP was performed in 27 patients and a right pyeloplasty in 14 patients. All procedures were performed using a transperitoneal approach. Our mean operative time was 135 min with a mean hospital stay of 1.5 days. The overall success rate for our series was 95%. Two patients had persistent SFU IV hydronephrosis requiring redo laparoscopic pyeloplasty and balloon dilatation, respectively. Four patients had post-operative complications including stent pain in 2 and non-obstructive small renal stone in 2. None of the patients less than a year of age had any complications. Residual hydronephrosis was identified in 29% of the patients.

Multiple authors have reported strong series with RALP (Table 1). A series of studies performed over the last decade show that when compared to open and laparoscopic pyeloplasty, the robotic assisted procedure has performed well in the treatment of UPJO (Table 2). These studies have shown comparable success rates with no statistically significance between the modalities.

**Table 1:** Series of reported robotic-assisted laparoscopic pyeloplasty cases.

**Author**	**Procedure**	**# of pts**	**Mean age (yrs)**	**Laterality UPJ**	**Approach**	**Mean op time (min)**	**Hospital stay (days)**	**Complications**	**Success rate (%)**
Kutikov et al. (15)	RALP	9	0.47	n/a	Transperitoneal	122.8	1.4	n/a	78
Avery et al. (16)	RALP	60	0.61	Bilateral (2)	Transperitoneal	232	1	7	91
Asensio et al. (17)	RALP	5	10.59	n/a	Transperitoneal	144	2.6	n/a	100
Olsen et al. (18)	RALP	65	7.9	n/a	Retroperitoneal	146	2	11	100
Minnillo et al. (19)	RALP	155	10.5	n/a	n/a	198.5	1.9	17	96
Singh et al. (20)	RALP	34	12	n/a	n/a	105	n/a	2	97
Atug et al. (21)	RALP	7	13	n/a	Transperitoneal	184	1.2	1	100
Franco et al. (22)	RALP	15	11.9	n/a	Transperitoneal	223	n/a	4	n/a
Perez-Brayfield	RALP	41	10.2	Right (14), Left (27)	Trans	135	1.5	5	95%

**Table 2:** Series of reported cases comparing open pyeloplasty, laparoscopic pyeloplasty, and robotic-assisted laparoscopic pyeloplasty.

**Author**	**Procedure (OP, LAP, RALP)**	**# of patients**	**Mean age (yrs)**	**Laterality UPJ**	**Approach**	**Mean operative time (min)**	**Hospital stay (days)**	**Complications**	**Success rate (%)**
Barbosa et al. (23)	RALP	58	7.2	Bilateral (10)	Transperitoneal	n/a	n/a	1	76.9
	OP	154	1.2	n/a	n/a	n/a	n/a	7	67.9
Yee et al. (24)	RALP	8	11.5	n/a	n/a	363	2.4	1	100
	OP	8	9.8	n/a	n/a	248	3.3	0	87.5
Subotic et al. (25)	OP	8	9.8	n/a	n/a	248	3.3	0	87.5
Lee et al. (26)	RALP	33	7.9	n/a	n/a	219	2.3	1	94
	OP	33	7.6	n/a	n/a	181	3.5	0	100
Song et al. (27)	OP	30	8.5	Right (8), Left (22)	Transperitoneal	192.5	6.6	4	96.7
	LP	30	10.5	Right (6), Left (24)	Transperitoneal	197.4	5.8	4	89.7
	RALP	10	11	Right (3), Left (7)	Transperitoneal	254.1	3.2	1	100
Cundy et al. (28)	OP vs. RALP	157,166	7, 8.1	n/a	n/a	RALP (Longer OT)	RALP (shorter HS)	5, 9	88.5, 87.3
	LP vs. RALP	97, 151	6.5, 10	n/a	n/a	no significant diff.	RALP (shorter HS)	10, 10	96.9, 99.3
Salö et al. (29)	OP	92	6.2	Right (38), Left (54)	n/a	167	4.4	25	92
	RALP	31	8.3	Right (10), Left (21)	Retro (15), Trans (16)	249	3.4	9	94

In comparison to open or laparoscopic pyeloplasty, robotic pyeloplasty typically exhibit shorter hospital stay and less use of medication for pain management following the procedure (4). The only consistent negative variable has been the longer operative times exhibited by the robotic approach as compared to other modalities. Operative times seems to improve in center with high volume and surgeon's experience.

## Costs and Considerations

Several studies have delved into the evaluation of costs of the treatment options for UPJO. Some studies have even suggested a 2.7 time increase in cost in RALP as compared to other modalities of UPJO (30). In 2017 Jacobs et al. (31) published a cost analysis study in adult patients showing fairly similar costs for open pyeloplasty ($22,421) as compared to minimally invasive pyeloplasty ($22,843). Varda and colleagues evaluated the national trends of UPJO treatment modalities in children including analysis of the available data on cost (32). They reported evidence of an increasing trend toward utilization of minimally invasive pyeloplasty over open pyeloplasty. In the study, minimally invasive modalities had an increased cost with a significant increase in price related to RALP. Operating room costs were by far the greatest contributor to costs, with robotic supplies being the largest contributor to the rising cost. For example, when comparing laparoscopic vs. robotic approaches there was an average increase in costs of over $3,000.

In another study, Varda et al. again demonstrated an increased utilization of the RALP in children (33). They showed that within a 12-year period there was a persistent higher cost when RALP was compared with open pyeloplasty. The increased cost in RALP over open pyeloplasty persisted as the cost of operating room equipment for robotic cases remained high even when considering the cost associated with longer hospital stays related to open surgery. High volumes of RALP may be required for institutions to profit from the procedures as total investment cost is divided between an increased number of procedures performed. An estimated three to five robotic cases per week are necessary to profit from robotic surgery, which is a clear limitation for pediatric centers no matter their size (34). Reaching the required number of cases needed will be a challenge to children's hospitals with low to mid volume RALP programs.

Based on our analysis and personal experience there appears to be clear evidence that there is in fact a higher cost to RALP as compared to open and laparoscopic approaches. Published data seems to suggest than even with shorter length of stay attributed to RALP as compared to other treatment modalities, the high cost of training, maintenance and materials point to a greater cost as compared to other modalities. In the near future innovation in technology, robotic market competition and market tendencies may see a further normalization of RALP costs that could be comparable to other treatment options.

### Parental Capital Gains

Other than the inherent cost analysis necessary for the evaluation and comparison of the treatment modalities of UPJO, there is also a further economic impact related to UPJO treatment as it pertains to parental gains/losses in the pediatric population. A 2011 study by Behan and colleagues evaluated the human capital gains associated to RALP in children (35). An evaluation of 44 patients most of which underwent RALP as compared to open approach was done retrospectively, in which indirect expenses to each procedure was estimated using already published financial models. Although parental work loss is sometimes used as the greatest variable to capital gains/loss other data was analyzed to evaluate the procedures. The results showed that the overall cost savings that are a result of decrease hospital length of stay for RALP may help compensate for the added operative costs previously alluded to. This study suggested that RP is associated with decreased lost parental wages and savings attributed to shorter length of stay, but the results are extremely dependent on the overall costs and amortization related to the robot. Prospective large center studies would be of great value to truly assess the impact of this variable in the treatment modalities of UPJO.

### Satisfaction

In the pediatric population satisfaction is not merely based on patient satisfaction and outcomes, but also related to parental satisfactions. Freilich et al. evaluated parental satisfaction based on a modified Glasgow Children's Benefit Inventory (36). Groups of open and RALP were compared based in most part to the responses of the questionnaire. Overall the results of the study showed that even when objective success of surgery were similar between groups (i.e., decreased hydronephrosis on imaging, improved renal scan measures), RALP was favored overall by parents. In regard to specific variables such as postoperative pain, speed to normal activity, speed of return to normal sports, surgery incision scar, impact of surgery on parental life, burden of postop visits/studies, and overall satisfaction, parents seemed to find a greater difference between actual results and expected results within the robotic wing. Based on this study there is increased satisfaction when RALP is undertaken especially in regards to cosmesis and recovery, but expectations as compared to actual results are almost always improved notwithstanding the type of treatment modality employed.

The effect of cosmesis takes greater impact when novel treatment techniques are utilized within the endoscopic treatment realm. For example, hidden incision endoscopic surgery (port sites at level of a Pfannenstiel incision) did show greater satisfaction from patients and parents in regard to cosmetic results in a series of 12 patients published by Gargollo in 2011 (37). In our experience endoscopic approaches are preferred by parents based on the reported considerations as well.

### Benefits vs. Risks

Apart from patient benefits like reduced pain, improved cosmetic results, shorter hospitalization, and rapid convalescence there are also added technical benefits to robotic surgery. Extensive published data exists on the benefits of magnified three-dimensional vision, the advantage of having an increased number of working arms, reduced tremors, and overall improved ergonomics. These qualities are an upside on robotic surgery when compared to both open and conventional laparoscopic approaches.

In a review of 5,400 laparoscopic cases performed Peters reported an overall complication rate of 5.4% (38). The greatest predictor of complication rate was surgeon experience.

Braga et al. published a systematic review and metanalysis of RALP vs. conventional laparoscopic approaches (39). RALP showed improved operative time reduction, and a significantly shorter stay at the hospital, but no statistical significance was found with regards to the rates of complications between the treatment modalities. The effects of reduced morbidity in robotic surgery, especially within the pediatric population, is also apparent due to a trend to its utilization in redo cases (40). Up to this point when compared to conventional laparoscopy there is no clear or definitive decrease in morbidity in RALP, especially in experienced hands.

### Future of Robotic Pyeloplasty

The future of RP seems to lie both on achieving greater utilization of the currently described technique as well as in the development of new techniques and technology. Single ports, smaller surgical sites, telesurgery, and hidden surgical incisions all seem to be in development and may show promise as they become more available.

Further miniaturization of robotic arms, especially in the form of table mounted systems, will allow for increased dexterity (4). Baek et al. published data regarding the use of 5 mm instruments for RALP in children of different ages (infants and non-infants). Utilization of smaller port sites allowed for safe intervention of RALP in infant children with similar results when compared to older children (41). Improvement in the 5 mm instrument and miniaturization of the robotic arms will facilitate RALP in the smaller infant patients (4).

Another area of particular interest is further development of force-feedback mechanisms to the surgeon that can compensate for the lack of tactile feedback in robotic cases. This in conjunction with newer technologies like virtual reality and augmented reality may not only change robotic surgery as a whole but may also improve education in robotic surgery including RALP.

## Conclusion

RALP is safe, effective, and well-accepted by surgeons, patients and their parents. There are real concerns regarding the longer operative times and cost associated with this procedure. As surgeons become better trained and have more experience with this technology operative time and associated cost should reduce significantly. Also, as more companies develop additional robotic technology, competition should produce more affordable robotic systems and instrumentations directly reducing the overall cost to the health systems. In the near future, RALP could become our new gold standard in the treatment of UPJO or at least be an equal to the open approach pyeloplasty.

## Author Contributions

RM-L, MP-M and MPB contributed in all aspects of the manuscript including research, writing, and editing of manuscript.

### Conflict of Interest Statement

The authors declare that the research was conducted in the absence of any commercial or financial relationships that could be construed as a potential conflict of interest.
